# Mutations in the Progesterone Receptor (PROGINS) May Reduce the Symptoms of Acute Hepatitis E and Protect Against Infection

**DOI:** 10.3389/fmicb.2019.02617

**Published:** 2019-11-07

**Authors:** Pedro López-López, Antonio Rivero-Juarez, Mario Frias, Isabel Machuca, Javier Caballero-Gómez, Israel Olivas, Angela Camacho, María de los Angeles Risalde, Ignacio García-Bocanegra, Antonio Rivero

**Affiliations:** ^1^Infectious Diseases Unit, Hospital Universitario Reina Sofía de Córdoba, Instituto Maimonides de Investigación Biomédica de Córdoba, University of Córdoba, Córdoba, Spain; ^2^Department of Animal Health, University of Córdoba, Córdoba, Spain

**Keywords:** PROGINS, progesterone-receptor, hepatitis E virus, HIV, susceptibility, symptoms, protect

## Abstract

**Background:**

Mutations in the progesterone receptor (PR) gene, PROGINS, have been studied in relation to hepatitis E virus (HEV) infection. Patients with the PROGINS gene may develop a worse clinical course of hepatitis E. The aim of our study was to evaluate the influence of PROGINS on the susceptibility to and the clinical course of HEV infection in HIV patients.

**Methods:**

This study included patients with HIV who were evaluated in previous prospective studies for the prevalence and incidence of HEV. The following three groups of patients were studied: (i) never infected, (ii) past infections, and (iii) recently infected. We determined the PR genotype to evaluate the proportion of patients who were homozygous for PROGINS according to HEV infection. We also compared the proportion of PROGINS carriers with a recent HEV infection according to their symptomatology.

**Results:**

In this study, 311 patients infected with HIV were included. Of those patients, 198 were homozygous wild type (63.7%), 91 were heterozygous (29.3%), and 22 were homozygous PROGINS (7.1%). We found that the homozygous PROGINS genotype in women was associated with a lower HEV seroprevalence. In addition, in patients with a recent HEV infection, none of those homozygous for PROGINS presented symptoms.

**Conclusion:**

The PROGINS mutation plays a protective role against HEV infection and is associated with subclinical infection in HIV-infected patients, particularly women.

## Introduction

Progesterone is a steroid hormone that downregulates immune system activity (Butts et al., 2007; Jones et al., 2010; Hall et al., 2017). *In vitro* studies have demonstrated that high levels of progesterone promote the downregulation of proinflammatory cytokines and chemokines (Arruvito et al., 2008; Devadas et al., 2018). Consequently, progesterone levels may promote susceptibility to different processes, as well as clinical features and evolution.

Mutations in the progesterone receptor (PR) can reduce the activity of the hormone progesterone (Romano et al., 2006, 2007). These mutations in the PR, called PROGINS (Rowe et al., 1995), consist of a 320-bp Alu insertion in intron G and two substitutions, one in exon 4 (V660L), and the other in exon 5 (H770H) (Romano et al., 2007). In the overall population, the frequencies of these mutations range from 0.07 to 0.26 (Modugno, 2004). Several studies have evaluated the role of PR gene polymorphisms and their associations in different pathologies including malignancies, where PROGINS could be a risk factor for uterine cancer and leiomyomas (Lee et al., 2010; Yuan et al., 2013; Gallegos-Arreola et al., 2015), or reproductive disorders in women that can cause infertility, where carrying the PROGINS gene is a risk factor for developing endometriosis (Costa et al., 2011; Silva and Moura, 2016).

PROGINS has also been reported to influence the activity of the immune system (Lhomme et al., 2016) and have an impact on the clinical features and evolution of viral infections. In this context, the PR has been studied in relation to hepatitis E virus (HEV) infection (Bose et al., 2011; Debes et al., 2018), where those with the PROGINS gene were observed to develop a worse clinical course of hepatitis E. The aim of our study was to evaluate the influence of PROGINS on the susceptibility to and the clinical course of HEV infection in HIV patients in an area with high prevalence and incidence of hepatitis E.

## Materials and Methods

### Patients

This study retrospectively included HIV patients who were evaluated in previous prospective studies of HEV prevalence and incidence carried out in the Province of Cordoba (Southern Spain) between 2012 through 2014 (Rivero-Juarez et al., 2015, 2017). Patient selection was based on a diagnosis of HEV infection and blood sample availability. Three groups of patients were created: (i) never infected, defined as IgG- and IgM-seronegative and aviremic; (ii) past infection, defined as IgG positive but negative for both IgM and HEV RNA; and (iii) recently infected, defined as IgM positive and/or HEV RNA positive. Data concerning the presence of symptoms associated with HEV infection as well as the epidemiological and clinical information of each patient were also collected in the recently infected subgroup. We followed the criteria for HEV screening as specified in clinical guidelines (European Association for the Study of the Liver, 2018; Rivero-Juarez et al., 2018).

### Variable Collection and Definition

The main outcome variable was infection with HEV, which was defined as past or recent infection (primary analysis). The secondary outcome variable was the presence of symptoms associated with HEV infection (secondary analysis).

### Anti-HEV IgG/IgM Serology and RT-PCR for the Detection of HEV

ELISA was used for the detection of anti-HEV IgG (Wantai HEV-IgG ELISA^®^; Beijing Wantai Biological Pharmacy Enterprise© LTD., Beijing, China) and anti-HEV IgM (Wantai HEV-IgM ELISA^®^; Beijing Wantai Biological Pharmacy Enterprise© LTD., Beijing, China). The ELISAs were carried out in accordance with the instructions provided by the manufacturer using a cut-off value of >1.1. The specimens with an absorbance value to cut-off ratio between 0.9 and 1.1 were considered borderline. All the anti-HEV IgG/IgM positive and borderline samples were confirmed by Western blot analysis (recomBlot HEV IgG/IgM^®^; Mikrogen Diagnostik GmbH, Neuried, Germany). RT-PCR for HEV RNA was performed on all patient samples (amplicube HEV^®^; Mikrogen Diagnostik GmbH, Neuried, Germany).

### Determination of PROGINS

The PR genotype was identified from retrospectively collected blood samples stored at −80°C until analysis. Genomic DNA was extracted from 200 μL of blood using the QIAamp DNA Blood Mini Kit (QIAgen, Hilden, Germany) and an automated procedure (QIAcube, QIAgen, Hilden, Germany). PCR was performed with MyTaq^TM^ DNA Polymerase (Bioline, Meridian Life Science, Memphis, TN, United States) together with the following primers (20 μM) used to detect intron G and identify the PR genotype: forward primer 5′-GCCTCTAAAATGAAAGGCAGAAAG-3′ and reverse primer 5′-GTATTTTCTTGCTAAATGTCTG-3′ (Agoulnik et al., 2004). The thermal profile was 95°C for 1 min followed by 35 cycles at 95°C for 15 s, 60°C for 15 s, and 72°C for 10 s. Electrophoresis was conducted with 10 μL of PCR products mixed with 1.6 μL of (6×) Gel Loading Dye, Blue (New England BioLabs) on a 2% agarose gel with 5 μL of ethidium bromide in a volume of 150 mL for 50 min at a constant voltage of 90 volts. The Tracklt 100-bp DNA ladder (Invitrogen, Burlington, ON, Canada) was used to identify the molecular weight of the bands in the agarose gel. The bands were visualized using the Molecular Imager Gel Doc XR System (BioRad, Hercules, CA, United States).

### Genotypic Classification of the Progesterone Receptor

**FIGURE 1:**
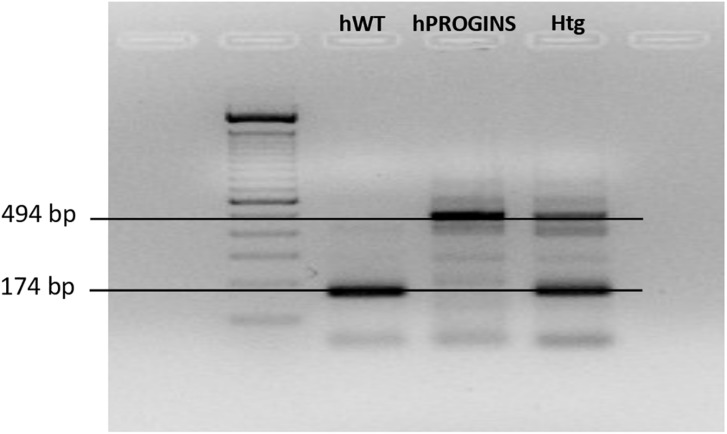
Identification of the progesterone receptor genotypes. hWT, homozygous wild type; hPROGINS, homozygous PROGINS; Htg, heterozygous; bp, base pairs.

We classified the patient genotypes by visualizing the different molecular weight bands in the gel. The 174-bp band corresponds to the wild-type genotype and the 494-bp band corresponds to the PROGINS genotype (Figure 1). The patients were classified prospectively as (i) homozygous wild-type; (ii) homozygous PROGINS; or (iii) heterozygous (Figure 1).

### Statistical Analysis

The prevalence of the PR genotypes in the study population was calculated. The categorical variables were expressed as the numbers of cases (percentages). The frequencies were compared using the χ2 test or Fisher’s exact test, and significance was set at a two-tailed *p*-value of less than 0.05. We used the χ2 test when the expected values of at least 80% of the cells in a 2 × 2 contingency table to be greater than 5. When these conditions are not verified, we compared the qualitative variables via the Fisher’s exact test. We have included this point in the section “Statistical Analysis.” The following formula was used to calculate the allele frequencies of the PR gene: 2^∗^N homozygous + N heterozygous/2^∗^N total. We first evaluated the proportion of patients who were homozygous for PROGINS or not according to HEV infection [never infected (group i) vs. infected (groups ii and iii)]. For the patients with a recent HEV infection (group iii), we also compared the proportion of individuals who were PROGINS carriers or not according to the presence of symptoms (symptomatic vs. asymptomatic). The analyses were carried out using SPSS statistical software package version 18.0 (IBM Corporation, Somers, NY, United States).

### Ethics Statement

This study was designed and performed according to the Helsinki Declaration. The local CEIC (Clinical Trial and Ethical Committee) approved the study protocol.

## Results

### Study Population

A total of 311 HIV-infected patients were included in the study: 191 (61.4%) males and 120 (38.6%) females. All patients were on antiretroviral therapy with undetectable viral load The distribution of patients according to HEV infection was the following: (i) never infected, 141 (45.3%); (ii) past infection, 131 (42.1%); and (iii) recent infection, 39 (12.6%). In terms of PR genotype, 198 were homozygous wild type (63.7%); 91 were heterozygous (29.3%); and 22 were homozygous PROGINS (7.1%) (Table 1). The allele frequencies were 0.78 for the wild type and 0.22 for the PROGINS allele, which are similar to other studies (McKenna et al., 1995; Runnebaum et al., 2001). In addition, the allelic frequencies for the PROGINS genotype were 0.21 and 0.22 in patients infected and never infected by HEV, respectively.

**TABLE 1:** Prevalence of HEV infection according to progesterone receptor genotype.

**Genotype**	**Never**	**Past**	**Recent**	
	**infected**	**infection**	**infection**	**Total**
	**(*N* = 141)**	**(*N* = 131)**	**(*N* = 39)**	**(*N* = 311)**
Homozygous wild-type	91 (64.5%)	81 (61.8%)	26 (66.7%)	198 (63.7%)
Heterozygous	41 (29.1%)	42 (32.1%)	8 (20.5%)	91 (29.3%)
Homozygous PROGINS	9 (6.4%)	8 (6.1%)	5 (12.8%)	22 (7.1%)

*HEV, hepatitis E virus; N, number of subjects.*

### The Association Between PROGINS and Risk for HEV Infection

The relationship between homozygous PROGINS and HEV infection in the total population was analyzed (Table 2). Among the homozygous PROGINS patients, 9 (40.9%) were never infected, while 132 of the non-homozygous PROGINS patients were never infected (45.7%) (*p* = 0.48) (Table 2). When the patients were classified by sex, an association was found between PROGINS and females in the never-infected group (Table 2).

**TABLE 2:** Comparative analysis of never-infected and HEV-infected patients homozygous for PROGINS in the total population and according to sex.

	**Homozygous**	**Never infected**	**Infected**	***p* value**
	**PROGINS**	**(*N* = 141)**	**(*N* = 170)**	
Total	No	132 (45.7%)	157 (54.3%)	0.480
	Yes	9 (40.9%)	13 (59.1%)	
Males	No	50 (28.6%)	125 (71.4%)	0.102
	Yes	3 (18.7%)	13 (81.3%)	
Females	No	82 (71.9%)	32 (28.1%)	<0.001
	Yes	6 (100%)	0 (0%)	

*HEV, hepatitis E virus; N, number of subjects.*

### The Association Between PROGINS and Symptomatic HEV Infection

Among the 39 patients with a recent HEV infection, 23 (59%) were asymptomatic and 16 (41%) showed symptomatic infection. The main symptoms identified in these patients were digestive alterations, nephropathies (chronic renal failure, and pyelonephritis), febrile syndrome, hepatic cytolysis, and cholestasis. In the overall analysis, none of the homozygous PROGINS patients presented symptoms (Table 3).

**TABLE 3:** Patients with a recent HEV infection: analysis of the total population and according to sex.

	**Homozygous**	**Asymptomatic**	**Symptomatic**	***p* value**
	**PROGINS**	**(*N* = 23)**	**(*N* = 16)**	
Total	No	18 (52.9%)	16 (47.1%)	<0.001
	Yes	5 (100%)	0 (0%)	
Men	No	13 (48.1%)	14 (51.9%)	<0.001
	Yes	5 (100%)	0 (0%)	
Women	No	5 (71.4%)	2 (28.6%)	NC^∗^
	Yes	0 (0%)	0 (0%)	

*^∗^Not calculable. HEV, hepatitis E virus; N, number of subjects.*

## Discussion

The results obtained in the present study demonstrate that the presence of the homozygous PROGINS genotype in women is associated with a lower HEV seroprevalence in HIV-infected individuals. Our findings suggest that this genotype reduces the susceptibility to HEV infection and is associated with a better clinical course of infection.

The function of the PR is associated with its binding to progesterone, a steroid hormone involved in immune system modulation (Jones et al., 2010). Previous studies have suggested that high levels of progesterone may be related to increased susceptibility to infection. Byrne et al. (2016) observed that women who used injectable progestin-only contraception were more susceptible to HIV infection. Furthermore, *in vitro* studies have suggested that progestins could reduce the secretion of proinflammatory cytokines and chemokines, alter the attraction of inflammatory cells, such as neutrophils and macrophages, and affect the apoptosis of natural killer cells (Arruvito et al., 2008; Huijbregts et al., 2014; Devadas et al., 2018; Preciado-Martínez et al., 2018). The role of progesterone in the immune system is also influenced by its binding to the receptor. In this context, PROGINS has been shown to alter the function of the progesterone hormone (Romano et al., 2006, 2007). Two different PR exist. The wild-type receptor is assumed to bind normally to progesterone, which means that the progesterone levels modulate actions in the immune system naturally. The PROGINS receptor that presents mutations binds more weakly to progesterone (Romano et al., 2007), thereby reducing progesterone activity regardless of blood hormone levels. Consequently, the relationship between the PROGINS receptor and low progesterone activity could reduce susceptibility to HEV infection.

With respect to the symptomatology, the majority of cases of HEV infection (90%) are generally asymptomatic and self-limiting (Dalton and Seghatchian, 2016); however, certain risk groups, such as cirrhotic patients, pregnant women and patients with HIV infection, follow a worse clinical course (Krain et al., 2014; Frias et al., 2018). In the prospective studies from which the population included in this study were derived, we prospectively evaluated the presence or absence of signs or symptoms of HEV infection in 39 patients who presented acute infection (Rivero-Juarez et al., 2015, 2017). Our study found an association between the PR genotype and the development of symptoms during HEV infection in which none of the HEV-infected patients with the homozygous PROGINS genotype presented symptoms.

Two previous studies found the opposite situation, namely that PROGINS mutations could be a risk factor for HEV infection (Bose et al., 2011; Debes et al., 2018). Bose et al. (2011) analyzed a population of pregnant women, which is very different from our population of HIV-infected patients, and Debes et al. (2018) analyzed only the seroprevalence of HEV infection associated with the presence of the PROGINS gene and they did not differentiate between sexes, a variable in which we found differences. In addition, both of these studies focused their analyses on those with PROGINS versus those who were not carriers of this allele, without specifying homozygosity. Our group took into account the patient’s genotype when analyzing the effect of PROGINS because heterozygous patients may have modulated responses through the presence of the wild-type allele. On this point, a meta-analysis performed by Pooley et al. (2006) suggested that the PROGINS gene has a codominant effect. Another study reported that the PROGINS allele has a gene dosage effect, whereby the expression of this gene is greater in individuals who present homozygosity (Wang-Gohrke et al., 2000). In addition, Alter et al. (2010) observed allelic dosage effects in transient tachypnea of the newborn, in which the PROGINS gene has a protective effect against this disease.

Our study also observed possible differences between males and females with respect to the effect of the homozygous PROGINS genotype on HEV infection. Prior studies have identified being male as a risk factor for HEV infection (Pineda et al., 2014; Zeng et al., 2017). The differences between the male and female sex hormone systems could also explain the differential immunological activity against HEV. According to Ghosh and Klein (2017), the disparity between men and women can be found in the development of immune responses to viral infections. Another study found that men have worse outcomes than women in infections such as hepatitis B and C associated with sex hormones (Ruggieri et al., 2018). In our study, the homozygous PROGINS genotype effect has more of an impact on women than men in terms of HEV infection or development of symptoms, which may explain why being male was identified as a risk factor for HEV infection.

A limitation of our study is the number of patients included. Due to the low frequency of the PROGINS allele in our population, the number of patients homozygous for this allele was relatively low, and we therefore had to assume that the allele frequencies were constant in the population to perform the statistical analysis.

## Conclusion

In conclusion, the PROGINS mutation in the PR gene plays a protective role against HEV infection and is associated with subclinical infection in HIV-infected patients, particularly women.

## Data Availability Statement

The raw datasets for this study can be found in the European Nucleotide Archive, PRJEB34891.

## Ethics Statement

The studies involving human participants were reviewed and approved by the Andalusian Clinical Trial and Ethical Committee. The patients/participants provided their written informed consent to participate in this study.

## Author Contributions

AR-J: full access to all the data in the study, takes responsibility for the integrity of the data and the accuracy of the data analysis, study concept and design, and obtain funding. IM, AC, and AR: patient recruitment. PL-L, MR, JC-G, IG-B, MF, IO, and AR-J sample collection and procedures. PL-L, MF, and AR-J: analysis and interpretation of the data. PL-L and AR-J drafting of the manuscript and statistical analysis. All authors: critical revision of the manuscript.

## Conflict of Interest

The authors declare that the research was conducted in the absence of any commercial or financial relationships that could be construed as a potential conflict of interest.
